# Mapping the *H2* resistance effective against *Globodera pallida* pathotype Pa1 in tetraploid potato

**DOI:** 10.1007/s00122-019-03278-4

**Published:** 2019-01-21

**Authors:** Shona M. Strachan, Miles R. Armstrong, Amanpreet Kaur, Kathryn M. Wright, Tze Yin Lim, Katie Baker, John Jones, Glenn Bryan, Vivian Blok, Ingo Hein

**Affiliations:** 1 The James Hutton Institute, CMS, Errol Road, Dundee, DD2 5DA UK; 2 School of Biology, University of St Andrews, North Haugh, St Andrews, KY16 9ST UK; 3 School of Life Sciences, Division of Plant Sciences at the JHI, University of Dundee, Dundee, DD2 5DA UK; 4 Thapar Institute of Engineering and Technology, Patiala, Punjab 147001 India; 5 Present Address: Columbia University, New York, NY 10027 USA; 6 Present Address: Synpromics, Edinburgh, EH25 9RG UK; 7 Scotland’s Rural College (SRUC), Kings Buildings, West Mains Road, Edinburgh, EH9 3JG UK

## Abstract

**Key message:**

The nematode resistance gene *H2* was mapped to the distal end of chromosome 5 in tetraploid potato.

**Abstract:**

The *H2* resistance gene, introduced into cultivated potatoes from the wild diploid species *Solanum multidissectum*, confers a high level of resistance to the Pa1 pathotype of the potato cyst nematode *Globodera pallida*. A cross between tetraploid *H2*-containing breeding clone P55/7 and susceptible potato variety Picasso yielded an F1 population that segregated approximately 1:1 for the resistance phenotype, which is consistent with a single dominant gene in a simplex configuration. Using genome reduction methodologies RenSeq and GenSeq, the segregating F1 population enabled the genetic characterisation of the resistance through a bulked segregant analysis. A diagnostic RenSeq analysis of the parents confirmed that the resistance in P55/7 cannot be explained by previously characterised resistance genes. Only the variety Picasso contained functionally characterised disease resistance genes *Rpi*-*R1*, *Rpi*-*R3a*, *Rpi*-*R3b* variant, *Gpa2* and *Rx*, which was independently confirmed through effector vacuum infiltration assays. RenSeq and GenSeq independently identified sequence polymorphisms linked to the *H2* resistance on the top end of potato chromosome 5. Allele-specific KASP markers further defined the locus containing the *H2* gene to a 4.7 Mb interval on the distal short arm of potato chromosome 5 and to positions that correspond to 1.4 MB and 6.1 MB in the potato reference genome.

**Electronic supplementary material:**

The online version of this article (10.1007/s00122-019-03278-4) contains supplementary material, which is available to authorized users.

## Introduction

The world population is predicted to reach eight billion by 2025 (Johnson 2001) and will bring with it a need to intensify crop production. Crop loss and damage due to plant parasitic nematodes is currently estimated to amount to $80 billion (£56 billion) annually, with the nematodes of the root-knot and cyst species being the most destructive (Nicol et al. 2010). In the UK alone, the potato cyst nematodes (PCN) *Globodera rostochiensis* and *G. pallida* are estimated to account for £26 million worth of crop damage per year (Twining et al. 2009). The increase in intensification of crop production that is required for the anticipated population growth brings with it an increase in the risk of pathogen and parasite infection. In addition, the requirement of long crop rotations due to the persistence of cysts in the soil and increasing restrictions on nematicide use makes breeding of resistant varieties key to mitigating the threat of PCN.

*Solanum tuberosum* ssp. *tuberosum* originates from South America, and cultivated potatoes introduced into Europe have a relatively narrow genetic base (Bryan et al. 2004). In many cases, effective resistances against major pathogens have been introduced into varieties from wild species. The first example of successful deployment of an effective major PCN resistance was the *H1* gene from *S. tuberosum* ssp. *andigena* (Ellenby 1954; Gebhardt et al. 1993). Although the *H1* gene has not been molecularly characterised, its introduction into varieties has been highly successful in controlling *G. rostochiensi*s infestations in, for example, the UK, where relatively little diversity exists in *G. rostochiensis* populations (Blok et al. 1997). However, this deployment has caused a shift in nematode populations towards *G. pallida*, which is not controlled by the *H1* resistance gene. Due to multiple *G. pallida* pathotypes being present in the UK and the lack of a single major resistance gene in potato that confers resistance to these pathotypes, very few potato varieties currently exist that contain sufficiently good resistances for sustained control of *G. pallida* and suppression of infection.

To date, eight resistances against nematodes have been mapped in tomato and potato (Davies and Elling 2015). Cloned and well-characterised resistances include *Hero* from *S. pimpinellifolium* (Ganal et al. 1995), *Mi1* from *S. peruvianum*, *Gro1*-*4* originating from *S. spegazzinii* (Paal et al. 2004) and *Gpa2* from *S. tuberosum* ssp. *andigena* (van der Voort et al. 1999). Importantly, these genes encode members of the nucleotide-binding, leucine-rich-repeat (NLR) family of resistances that form part of the STAND (Signal Transduction ATPase with Numerous Domains) protein family (Lukasik and Takken 2009). Since NLRs also control other pathogens of *Solanaceae* such as viruses and oomycetes, significant efforts have focused on this gene family with the aim of identifying and characterising novel pathogen resistances (Jupe et al. 2012). The activation of NLRs, which are overall conserved in their structure (Urbach and Ausubel 2017), typically elicits a complex resistance response including hormones which play a pivotal role in pathogen defence within the plant kingdom (Crampton et al. 2009).

Over the last two decades, the cost of whole genome sequencing has steadily decreased, enabling the analysis of large plant genomes. The potato reference genome of the doubled-monoploid *S. tuberosum* group Phureja clone DM1-3 516 R44 (DM) was published in 2011 (PGSC 2011) and has enabled an in-depth analysis of NLR gene diversity and organisation within the potato genome (Jupe et al. 2012). The development of NLR-specific enrichment sequencing (RenSeq) has facilitated a more comprehensive NLR gene annotation (Jupe et al. 2013). Furthermore, RenSeq, which targets all 755 described NLRs in potato, has been successfully used to map and/or identify functional NLRs against late blight (Chen et al. 2018; Jupe et al. 2013; Witek et al. 2016). Used as a diagnostic tool in wild species or varieties, and referred to as dRenSeq, the technology allows for the rapid assessment as to whether a resistance phenotype is based on already characterised resistances or a hitherto unknown gene (Armstrong et al. 2018; Chen et al. 2018; Jiang et al. 2018; Van Weymers et al. 2016). To compliment RenSeq-based mapping, we previously developed GenSeq, a targeted enrichment sequencing approach of 1980 single or low-copy number genes that can be placed on the individual potato chromosomes with high confidence (Chen et al. 2018).

Here, we present the successful application of RenSeq and GenSeq enrichment sequencing (Chen et al. 2018) to genetically characterise the *H2* resistance which originates from the wild species *S. multidissectum* (Dunnett 1961) in a tetraploid background. *H2* provides resistance against *G. pallida* pathotype Pa1 while also conferring a lower level of control to pathotypes Pa2/3 (Blok and Phillips 2012). The two enrichment sequencing approaches, in combination with a bulked segregant analysis, independently mapped the *H2* resistance to the short arm of chromosome 5.

## Materials and methods

### Potato material

A cross between the susceptible variety Picasso and the resistant clone P55/7 yielded true seeds from which an initial F1 population of 192 progeny clones was used for the phenotyping of the *G. pallida H2* resistance.

### Resistance screen using *G. pallida* Pa1

Plants underwent two independent cycles of infection assays with Pa1 cysts to determine whether they were resistant or susceptible. In addition to the resistant and susceptible parents P55/7 and Picasso, 192 F1 progeny clones were grown and phenotyped over 2 years. In year one, 154 F1 progeny plants were assessed, while in year two all 192 F1 progeny plants were phenotyped. For both cycles of phenotyping, each progeny clone was propagated to generate three replicated clonal plants which were each infected with 15 ± 2 Pa1 cysts, respectively. Progeny plants were established in root trainers and left to grow for 7 days before infection with Pa1 cysts. Infected plants were left for 8 weeks before the root trainers were opened to expose the root system. Visible females were counted from all sides of the root system. Average scores were calculated from technical replicates and, where possible, from year one and two replicates. Using the average scores of the parental controls (resistant P55/7: average < 1; susceptible Picasso: average > 18), progeny clones were classed as very resistant (< 1 female) or susceptible (> 18 females). Across the 2 years of infection assays, 57 clones were phenotyped in both technical and biological replicates. Any clone that displayed contradictory phenotypes between the two assays was removed, and only the 20 most resistant and susceptible progeny plants were selected for further analysis. Where insufficient progeny plants with contrasting phenotypes were identified in the combined year one and two phenotyping assays, clones with consistent phenotypes in year two were chosen as inoculations yielded the most robust root infections.

### Genome enrichment and read mapping

DNA extraction from potato leaves along with GenSeq and RenSeq sequencing library preparation was undertaken according to Chen et al. (2018). The bait libraries for GenSeq and RenSeq have been described by Chen et al. (2018) and can be obtained from http://solanum.hutton.ac.uk. Paired-end Illumina MiSeq reads were first checked with FastQC (Andrews 2010) and then quality and adapter trimmed with cutadapt v1.9.1 (Aronesty 2011) to a minimum base quality of 20. All reads are publicly available and have been submitted to the European Nucleotide Archive (https://www.ebi.ac.uk/ena) with the ENA accession number PRJEB28455.

For the genetic mapping of the resistance, trimmed RenSeq or GenSeq reads were mapped to the DM potato reference genome (v4.03) (PGSC 2011; Sharma et al. 2013) using Bowtie2 v2.2.1 (Langmead and Salzberg 2012) in very-sensitive end-to-end mode. Discordant and mixed mappings were disabled, and the maximum insert was set to 1000 bp. Three score-min parameters were used in different mapping runs: “L, − 0.12, − 0.12”, “L, − 0.18, − 0.18” and “L, − 0.3, − 0.3”, approximately equal to 2%, 3%, and 5% mismatch rates, respectively. The resulting BAM files were sorted, merged and indexed using SAMtools (Li et al. 2009). Pile-up files were generated for the bulks and parents using SAMtools mpileup with default settings and piped into VarScan mpileup2snp (Koboldt et al. 2012) with—strand-filter 0 and—output-vcf 1 for variant calling.

For the dRenSeq analysis, RenSeq reads were mapped against a bespoke set of functionally characterised NLRs, including their 5′ and 3′ flanking region (Armstrong et al. 2018). In addition to the references described by Armstrong et al. (2018), additional NLRs *Gro1*-*4* [AY196151] (Paal et al. 2004); *Hero* [AJ457051] (Ganal et al. 1995); *Mi1.1* [AF039681] and *Mi1.2* [AF039682] (Milligan et al. 1998); *Rpi*-*abpt* [FJ536324.1] (Lokossou et al. 2009); *Rpi*-*amr* [KT373889] (Witek et al. 2016); and *Rpi*-*Ph*-*3* [KJ563933.1] (Zhang et al. 2014) were included. Only paired-end reads were mapped using the score-min parameter L, − 0.01, − 0.01. This results in a mismatch penalty of 5 per 250 bp to only map reads without any high-quality polymorphisms compared to the reference set. Due to the high nucleotide similarity of NB-LRR sequences, this enabled mapping to a maximum of ten positions (− k 10). The resulting BAM file was aligned and indexed using SAMtools (Li et al. 2009) v1.3.1. Read depth and coverage were calculated using BEDTools (Quinlan and Hall 2010). Data were subsequently transformed and plotted using a custom script in R Studio (RStudio 2014).

### SNP filtering

SNPs were filtered using a custom Java code to retain informative SNPs present in both bulks and parents. SNPs were filtered based on expected allele ratios for susceptibility/resistance plants and bulks (susceptible: rrrr; resistant: Rrrr). To be retained, each informative SNP had to be supported by a minimum of 50 RenSeq or GenSeq reads, respectively, and be present in an alternate allele frequency reflecting the expected genotype. Using ‘*r*’ as a reference, a 0–5% alternate allele frequency was selected in susceptible plants and 20–30% alternate allele for resistant plants. Conversely, using ‘*R*’ as a reference, SNPs that displayed 95–100% alternate allele frequency in susceptible plants and 70–80% alternate allele frequency in resistant plants were filtered. BEDTools intersect (Quinlan and Hall 2010) was used to extract SNPs present in both bulks and parents (informative SNPs) and to relate the informative SNP locations to the PGSC v3.4 gene annotations. The number of parental, bulk and informative SNPs from RenSeq and GenSeq was plotted in 1 Mb bins across each chromosome and visualised using R (R Core 2018).

### Read coverage and on-target estimation

Percentage of on-target reads was calculated as the proportion of reads mapping to annotated GenSeq or RenSeq target regions within the DM reference genome. Intersecting these regions (± 1000 bp) against the mapped reads using BEDTools gave the number of on-target reads. These on-target reads were then displayed as a proportion of the total number of mapped reads. Read coverage to target regions was calculated by dividing the total number of GenSeq/RenSeq mapped base pairs (± 1000 bp) by the total length of the GenSeq/RenSeq genes (+ 2000 bp per gene).

### KASP assays

Allele-specific KASP markers were designed against informative chromosome 5 SNPs identified through RenSeq and GenSeq under different mapping mismatch rates. Sequences flanking the informative SNPs 50 bp upstream and downstream were extracted and used in a MEGABLAST against the DM genome v4.03 via the BLAST + command line application (Camacho et al. 2009) at default settings to establish the potential specificity. In total, 11 KASP designs had no off-target BLAST hits compared to the DM genome (defined as > 95% sequence identity over at least 28 bp) and were advanced for KASP marker synthesis (Table S1).

SNP markers were designed according to LGC Genomics specification. Assays were carried out in 8.11 μl, 4 μl gDNA (20 ng/μl), 4 μl KASP Master Mix (LGC Genomics) and 0.11 μl KASP primer mix (KASP designed oligos). PCR reactions were undertaken using a StepOnePlus thermocycler with the following conditions: 2 min at 20 °C, ten cycles of 15 min at 94 °C, 20 s at 94 °C, 1 min at 62 °C (decreasing by 0.7 °C per cycle), 32 cycles of 20 s at 94 °C, 1 min at 55 °C and 2 min at 20 °C.

### Vacuum infiltration of potato leaves

Recombinant *Agrobacterium tumefaciens* was grown overnight in 10 ml LB medium. Samples were centrifuged and resuspended in 5 ml Agro-mix (10 mM MES, 10 mM MgCl_2_, 150 µM acetosyringone) and diluted with an OD_600nm_ of 0.5. Samples were then kept in the dark and left to incubate on a shaking plate at room temperature for 3 h. Potato leaves were subsequently vacuum infiltrated as described previously (Wei et al. 2007).

## Results

### Segregation of the *H2* resistance in Picasso × P55/7 F1 population suggests the presence of a single dominant *R* gene

**Fig. 1:**
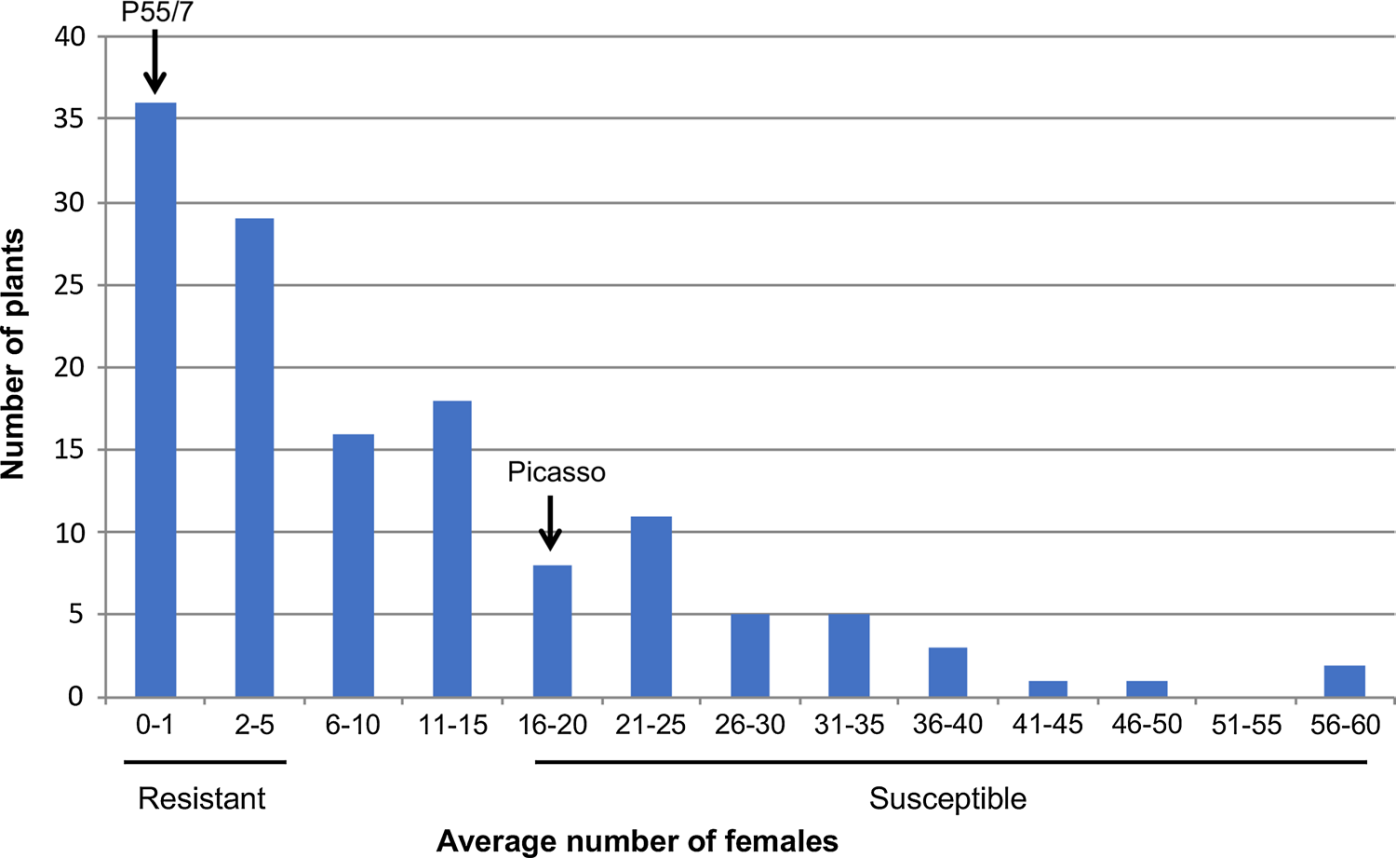
Histogram showing the distribution of average infection levels of 154 Picasso × P55/7 F1 progeny plants based on two independent replicates of six clones. The plants segregated in a near 1:1 ratio for resistance and susceptibility. The arrows indicate the level of infection in resistant parent P55/7 and susceptible variety Picasso. The x-axis shows the average number of females on infected plants and the y-axis the number of F1 progeny

A cross between the *H2*-containing resistant clone P55/7 and the susceptible variety Picasso was assessed for segregation of *G. pallida* resistance. Out of the 192 F1 progeny, 154 plants were successfully assessed in two independent phenotypic screens using three plants per genotype. In screen 1, 11 progeny plants were scored as very resistant (average ≤ 1 females), 23 as very susceptible (average ≥ 17 females) and 28 clones as intermediately susceptible (average > 1, < 17). For screen 2, 27 clones were scored as very resistant (≤ 1 females), 32 progeny clones as susceptible (≥ 18 females) and 69 as intermediately susceptible (> 1, < 18) (Table S2). Based on the mean number of females present across the three biological replicates and two independent experimental repeats, the distribution of the plant resistance was plotted (Fig. 1). The progeny segregated with a 0.8:1 (resistant: susceptible) ratio (*χ*^2^ = 0.04, *p* > 0.84) which is close to the 1:1 ratio expected for a simplex dominant resistance allele segregating in a tetraploid cross (Rrrr × rrrr). The 20 most consistently very resistant progeny (scoring ≤ 1 female) and 20 consistently very susceptible progeny plants (scoring ≥ 18 females) were selected for further genetic analysis using a bulk-segregant analysis approach.

### dRenSeq analysis reveals *H2* function is independent of previously characterised NLRs

**Fig. 2:**
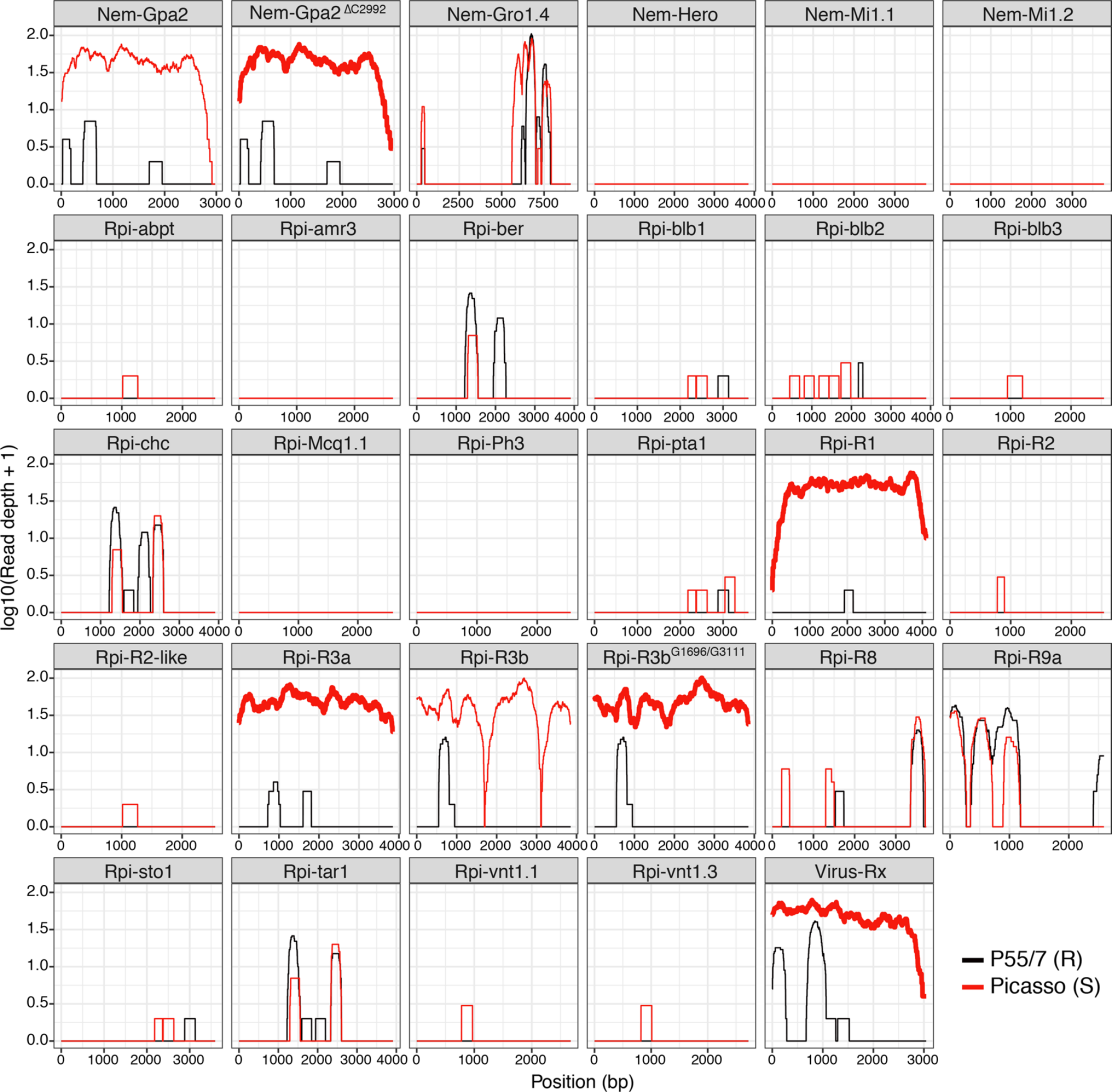
dRenSeq analysis of parent varieties Picasso (red) and P55/7 (black). RenSeq-derived reads are mapped against a reference set of 29 known NB-LRR genes in very-sensitive mode. Each box represents an entire NLR coding sequence from the start codon to the stop codon (x-axis). The y-axis reveals the coverage of the NLRs on a log scale. NLRs with full coding sequence representation are in bold

To evaluate whether the resistance phenotype in the resistant clone P55/7 and segregating within the F1 progeny could be associated with an already characterised NLR, diagnostic RenSeq (dRenSeq) analysis was conducted. RenSeq-enriched paired-end reads were mapped against a panel of known functional NLRs including the nematode resistance genes *Gpa2*, *Gro1.4*, *Hero* and *Mi1.1/1.2* to assess their presence within Picasso and P55/7. For a gene to be considered **‘**present’ within a variety, the sequence had to be identical when compared to the reference gene coding sequence. DRenSeq analysis in susceptible Picasso identified NLRs *Gpa2*, *P. infestans R* genes (*Rpi*) *Rpi*-*R1*, *Rpi*-*R3a*, *Rpi*-*R3b* variant *Rpi*-*R3b*^*G1696/G3111*^ described by Armstrong et al. (2018) and the NLR *Rx* effective against potato virus X. In contrast, no known characterised NLR was identified in the breeding clone P55/7 (Fig. 2).

To verify the dRenSeq results, *in planta* testing was conducted to confirm the presence and functionality of identified NLRs in Picasso and their absence in P55/7. Cognate avirulence (*Avr*) genes of selected NLRs were cloned into a suitable binary vector and delivered via *A. tumefaciens* vacuum infiltration into young Picasso and P55/7 leaves to assess recognition via cell death responses (Fig. S1). In perfect agreement with dRenSeq, transient expression of *Avr* genes *RBP1* (recognised by *Gpa2* (Sacco et al. 2009)), *Avr3a*^*KI*^ (recognised by *Rpi*-*R3a* (Bos et al. 2009; Chapman et al. 2014)), *Avr3b* (recognised by *Rpi*-*R3b* (Li et al. 2011)) and *Cp* (recognised by *Rx* (Bendahmane et al. 1995)) in Picasso but not in P55/7 elicited a cell death response. Transient expression of positive control CRN2 (Haas et al. 2009) elicited a strong and consistent response in both Picasso and P55/7, whereas no response was elicited by the control constructs (eGFP and *Avr2* (recognised by *Rpi*-*R2*)(Gilroy et al. 2011)).

DRenSeq in combination with resistance tests confirmed that *Gpa2* from Picasso (which segregated in the F1 population and was found in both bulked resistant and bulked susceptible progeny clones—data not shown) does not control *G. pallida* pathotype Pa1. Thereby, dRenSeq provides evidence that the *H2* resistance is based on a hitherto unknown gene and cannot be explained by the presence of previously characterised NLRs.

### RenSeq-based mapping places *H2* on chromosome 5

To genetically characterise the *H2* resistance, we conducted a bulked segregant analysis using individually indexed parents, bulked susceptible and bulked resistant samples (Table S2). The individually indexed genomic DNA samples from both parents and the two bulks were first subjected to RenSeq-based enrichment sequencing, which specifically targets NLRs (Jupe et al. 2013).

From a total of 8,511,314 paired-end reads obtained from RenSeq, 8,477,489 passed read trimming. The on-target mapping rate to the DM reference ranged from 30.37 to 61.86% (Table 1). Based on the phenotypic segregation ratio of nearly 1:1, which suggests that a single gene in a simplex configuration underpins the *H2* resistance, SNPs conforming to the expected ratios (‘Rrrr’ in P55/7 and resistant bulk as well as ‘rrrr’ in Picasso and susceptible bulks) were retained. This analysis was independently conducted at 2%, 3% and 5% mapping mismatch rates to allow for sequence variations compared to the DM reference genome.

**Table 1:** Details of reads for both GenSeq and RenSeq enrichments

	Sample		Total reads	Reads mapped to DM target regions at different mismatch rates					
				2%		3%		5%	
				Mapped reads	% of total reads	Mapped reads	% of total reads	Mapped reads	% of total reads
RenSeq	Parents	Picasso	4,620,522	1,403,248	30.37	1,472,010	42.74	2,696,198	58.35
		P55/7	3,444,110	1,088,722	31.61	1,920,970	41.57	2,041,726	59.28
	Bulks	Resistant	4,463,384	1,528,228	34.24	2,028,332	45.44	2,750,726	61.63
		Susceptible	4,426,962	1,488,068	33.61	1,955,120	44.16	2,738,474	61.86
GenSeq	Parents	Picasso	6,838,428	3,090,230	45.19	3,943,570	57.66	4,899,600	71.65
		P55/7	5,866,422	2,631,058	44.85	3,392,730	57.83	4,236,880	72.2
	Bulks	Resistant	6,087,178	2,919,814	47.97	3,658,688	60.1	4,464,938	73.35
		Susceptible	4,476,272	2,208,516	49.34	2,770,088	61.88	3,357,480	75.01

The number of reads which passed trimming for Picasso (rrrr), P55/7 (Rrrr), resistant progeny and susceptible progeny for each of the mismatch rates is detailed, as well as the percentage of reads which mapped to the DM target regions at each mismatch rate

**Fig. 3:**
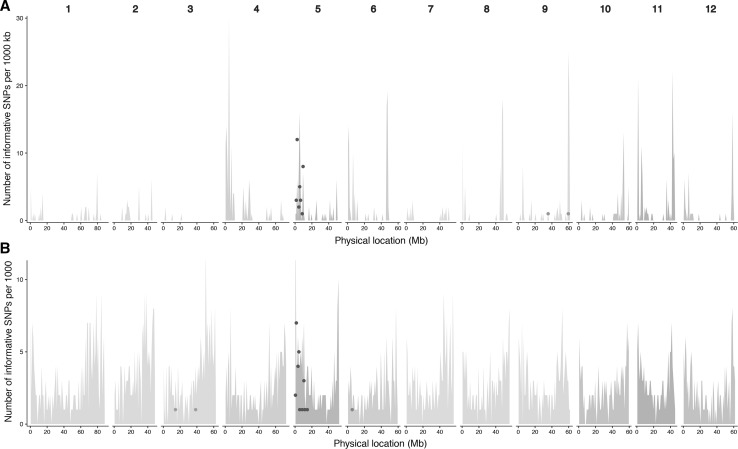
Graphical representation of SNPs linked to *H2* following RenSeq (**a**) and GenSeq analysis (**b**). Potato chromosomes 1–12 are depicted on the x-axis, and the numbers of informative SNPs within a 1 MB interval are shown as dots. **a** A total of 36 informative SNPs were identified during RenSeq with 34 SNPs being present on chromosome 5 and 2 SNPs on chromosome 9. **b** A total of 28 informative SNPs were identified during GenSeq analysis with 25 being present on chromosome 5, two SNPs on chromosome 3 and a single SNP on chromosome 6. Shaded in the background are the numbers of genes that were assessed at each locus and represent in this case the position of known NB-LRRs (RenSeq) or single/low-copy genes (GenSeq) used for the bait library designs

At a 3% mismatch rate, 3314 SNPs were identified between the parents Picasso and P55/7 that conformed to the expected allele frequency. In the bulks, 106 SNPs passed the filtering conditions expected for susceptible as well as resistant progeny. Of those SNPs, 36 were independently identified at the expected allele frequency in the parents and the bulks (Fig. 3a). The 36 SNPs correspond to 15 NLRs in the DM genome. More than 94% of these SNPs (34 out of 36) reside in an 8.1 MB interval on potato chromosome 5 and can be attributed to 13 NLRs. The remaining two SNPs correspond to two NLRs on chromosome 9 (Table 2).

**Table 2:** RenSeq informative SNPs identified at 3% mismatch rate

Chromosome	Start	Stop	Gene ID	Number of SNPs
5	1,500,545	1,506,500	ID = PGSC0003DMG400025099	3
5	2,063,328	2,066,456	ID = PGSC0003DMG400000813	1
5	2,075,262	2,079,628	ID = RDC0001NLR0074	1
5	2,185,980	2,190,589	ID = RDC0001NLR0075	2
5	2,201,139	2,204,777	ID = RDC0001NLR0076	8
5	4,227,604	4,230,353	ID = PGSC0003DMG400030497	1
5	4,589,149	4,595,717	ID = PGSC0003DMG400018428	1
5	5,469,503	5,473,373	ID = PGSC0003DMG400023062	2
5	5,723,483	5,731,577	ID = PGSC0003DMG400025611	3
5	6,506,321	6,508,868	ID = RDC0001NLR0090	1
5	6,528,097	6,537,250	ID = PGSC0003DMG401022603	2
5	8,619,648	8,627,296	ID = PGSC0003DMG400013506	1
5	9,635,954	9,642,604	ID = RDC0001NLR0098	8
9	35,461,259	35,467,442	ID = RDC0001NLR0212	1
9	59,518,316	59,519,194	ID = PGSC0003DMG400024366	1

Column 1 denotes the chromosome where the SNP(s) was identified, columns 2 and 3 give the start and stop positions of the gene, column 4 gives the gene ID and column 5 displays the number of SNPs found within the gene

Relaxing the mapping mismatch rates to 5% or increasing the stringency to 2% yielded 6192 SNPs and 1602 SNPs in the parents, respectively. In the bulks and parents, 66 and 10 SNPs passed the filtering criteria at these mismatch rates, respectively (Tables S3 and S4). In agreement with the 3% mismatch rate, most SNPs that passed the filtering criteria independently in the parents and bulks are associated with NLRs from chromosome 5. At the 5% mismatch rate, 55/66 SNPs (> 83%) can be attributed to 16 NLRs that reside in the same interval identified at the 3% mismatch rate between positions 1.5–9.6 MB (Table S3). At a 2% mismatch rate, 70% of SNPs are associated with a similar interval on potato chromosome 5 (Table S4).

### GenSeq data confirms position of *H2* on chromosome 5

To independently validate the RenSeq-inferred mapping position of *H2* to chromosome 5, the indexed parents and bulks used for RenSeq were also subjected to GenSeq-based enrichment. The on-target rate for GenSeq reads was comparable to RenSeq and ranged from 44.85 to 75.01%. As with the RenSeq sequencing data, GenSeq-derived reads were mapped to the DM genome at different mismatch thresholds including 2%, 3% and 5% (Table 1). Filtering for informative SNPs, which were independently identified in the bulks and parents, yielded 18 SNPs at a 2% mismatch rate (Table S5), 28 SNPs at a 3% mismatch rate (Table 3) and 54 SNPs at a 5% mismatch rate (Table S6). Importantly, the majority of these SNPs, ranging from 87% at a 5% mismatch rate, 89% at the 3% mismatch rate to over 94% at the 2% mismatch rate, correspond to genes associated with the top end of chromosome 5 (Fig. 3b). Combining the data from RenSeq and GenSeq analyses independently corroborated the mapping position of the *H2* resistance to an approximately 11 MB interval on potato chromosome 5 (Fig. 3a and b).

**Table 3:** GenSeq informative SNPs identified at 3% mismatch

Chromosome	Start	Stop	Gene ID	Number of SNPs
3	14,879,240	14,879,866	ID = PGSC0003DMG400040532	1
3	38,314,819	38,321,395	ID = PGSC0003DMG400018852	1
5	644,928	648,054	ID = PGSC0003DMG401028313	1
5	668,859	673,110	ID = PGSC0003DMG400028364	1
5	1,415,273	1,419,957	ID = PGSC0003DMG400025119	1
5	1,437,168	1,441,274	ID = PGSC0003DMG400025121	6
5	2,997,356	3,001,120	ID = PGSC0003DMG400014571	2
5	3,357,219	3,357,723	ID = PGSC0003DMG400030589	1
5	3,710,910	3,715,061	ID = PGSC0003DMG400030518	1
5	4,173,679	4,174,911	ID = PGSC0003DMG400030500	4
5	4,484,319	4,492,247	ID = PGSC0003DMG400018405	1
5	5,028,894	5,038,966	ID = PGSC0003DMG400031261	1
5	8,383,814	8,387,263	ID = PGSC0003DMG400030998	1
5	10,524,338	10,532,794	ID = PGSC0003DMG400018598	1
5	10,714,245	10,719,910	ID = PGSC0003DMG400011723	2
5	11,252,622	11,256,056	ID = PGSC0003DMG400010739	1
5	14,418,005	14,425,294	ID = PGSC0003DMG400034313	1
6	5,041,666	5,044,538	ID = PGSC0003DMG402004406	1

Column denotes the chromosome where the SNP resides. Columns 2 and 3 give the start and end positions of the gene containing the SNP. Column 4 contains the gene name, and column 5 shows the number of SNPs present in each gene

### SNP-based KASP markers from RenSeq and GenSeq analyses define the *H2* resistance locus to a 4.7 Mb interval on chromosome 5

To validate the linkage of RenSeq- and GenSeq-derived SNPs to *H2* and to further define the mapping interval on chromosome 5, SNPs were converted into allele-specific KASP markers. In total, 11 KASP markers were designed based on nine GenSeq SNPs and two RenSeq SNPs (Table S1). All RenSeq-derived SNPs and seven GenSeq-based polymorphisms were based on the 3% mismatch threshold. In addition, one SNP from the GenSeq analysis conducted at a 5% mismatch rate (PGSC0003DMG400018411) and one SNP from the 2% mismatch analysis (PGSC0003DMG400017618) were included. The marker designations reflect their positions within the DM genome version 4.3. For example, marker ST04_03ch05_1416331 is located on chromosome 5 at position 1416,331 bp within the DM reference genome.

**Fig. 4:**
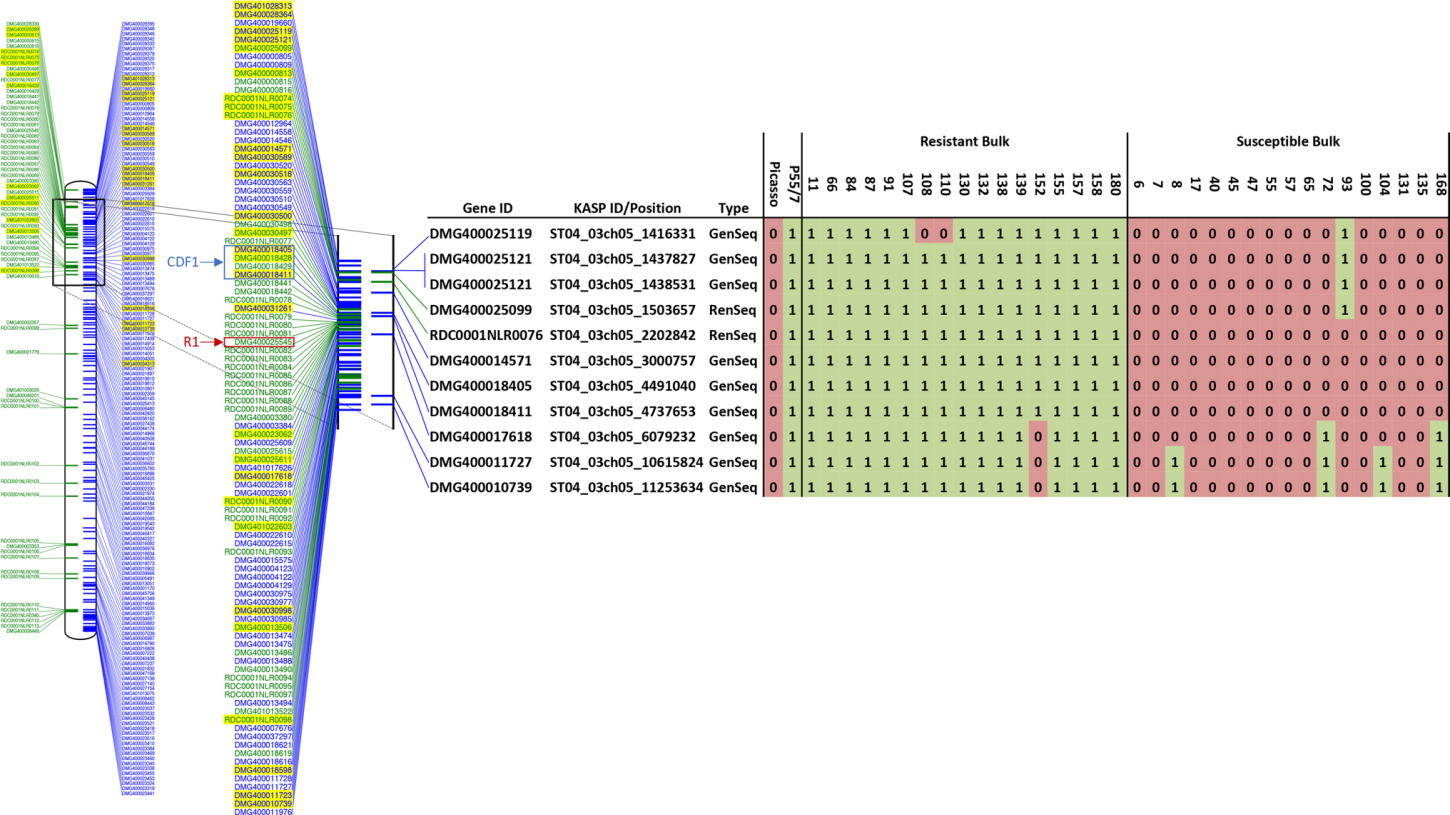
Mapping of *H2* to potato chromosome 5. Highlighted in the chromosome 5 ideograms are RenSeq genes in green and GenSeq genes in blue. The image on the left depicts the entire potato chromosome 5 and the adjacent image a close-up of the target region. Genes linked to *H2* through significant SNPs are highlighted in yellow. The relative position of the late blight resistance gene *R1* (Ballvora et al. 2002)—homologous to PGSC0003DMG400025545— is highlighted in red. The position of Cycling Dof Factor1 (CDF1) (Hannapel et al. 2017)—PGSC0003DMG400018408—is indicated in blue. Genes selected for linkage analysis through KASP markers are depicted alongside the gene ID, KASP ID/position according to DM and the marker type (RenSeq or GenSeq). The graphical genotypes of parents and individual progeny clones are shown. Samples designated with a 1 contained the same allele as the resistant parent (P55/7), whereas samples designated as 0 contained the same allele as the susceptible parent (Picasso)

The F1 progeny clones used to generate the bulked resistant and susceptible pools were individually analysed using GenSeq- and RenSeq-derived KASP markers. Using the allele discrimination output from each KASP assay, a graphical genotype could be assigned that corresponds to the ‘*R*’ or ‘*r*’ alleles, respectively. Each allele assessed with a KASP marker was assigned a ‘1’ (green) if it originated from the resistant parent (P55/7), while those designated ‘0’ (red) showed the same allele as the susceptible parent (Picasso) (Fig. 4). Arranging the phenotypes of the individual progeny clones with the KASP marker-derived genotype revealed three recombination events in resistant F1 clones 108, 110 and 152 as well as five recombinations in susceptible clones 8, 72, 93, 104 and 168. The most informative markers for delimiting the *H2* interval were RenSeq marker ST04_03ch05_1503657 which is based on NLR PGSC0003DMG400025099 and GenSeq marker ST04_03ch05_6079232 detecting an informative SNP in PGSC0003DMG400017618. These markers identified one recombination event (susceptible clone 93) and three recombination events (resistant clone 152 and susceptible clones 72 and 168), respectively, and thereby reduced the *H2* locus to 4.7 Mb. Importantly, the marker order as inferred by the DM potato reference genome is conserved in this interval as no apparent double recombination events are observed in either parent or F1 progeny.

## Discussion

Globally, nematodes are found in every ecological niche and those specialised as plant parasitic nematodes pose a serious and continuous threat to global crop production (Masler 2013). Of particular importance to potato are the cyst nematodes *G. pallida* and *G. rostochiensis* but root-knot nematodes such as *Meloidogyne incognita*, *M. arenaria* and *M. javanica*, *M. hapla*, *M. fallax* and *M. chitwoodi* also threaten *Solanaceous* plants (Caromel and Gebhardt 2011). *Globodera* ssp. have formed a very close relationship with *Solanum* ssp. which indicates a long process of coevolution between the nematodes and their hosts. Indeed, it has been speculated that the Incas introduced 6–8 years potato crop rotation in South America to mitigate PCN (Picard et al. 2007). Several distinct pathotypes (subpopulations) have been reported for *G. rostochiensis* and *G. pallida* that are differentiated based on their ability to infect *Solanum* ssp. containing different resistances (Kort et al. 1977). Compared to other diseases such as late blight caused by the oomycete pathogen *P. infestans*, very few resistances effective against PCN have been characterised.

To date, no NLR has been discovered which confers complete resistance to all European pathotypes of *G. pallida* (Pa1, Pa2/3) and current crop protection relies therefore on the application of chemicals or limited utilisation of quantitative resistances. Breeding for quantitative resistance is, however, extremely challenging due to the complexities of incorporating multiple genes with minor effects simultaneously into commercially viable varieties. Conversely, reliance on a single major NLR in the face of a variable pathogen population may mean that the effectiveness of this resistance is short-lived (van der Voort et al. 2000). Pyramiding multiple complimentary NLR genes is a desirable approach to prolong the longevity of NLRs. With regard to PCN resistance, the *H2* gene is a good candidate due to its strong resistance phenotype to *G. pallida* Pa1 and the partial resistance it provides to Pa2/3. In this study, we successfully mapped the *H2* resistance to a 4.7 MB interval on potato chromosome 5. This is a first step towards the cloning of the *H2* resistance gene which could then be deployed in varieties and combined through dRenSeq technology with complimentary resistances such as *H3* or *GpaV* and others as and when they are characterised (Armstrong et al. 2018).

The mapping of *H2* was carried out in a tetraploid background which is, compared to a diploid configuration, more complex. Nevertheless, genome reduction in combination with bulked segregant analysis not only identified sequence polymorphisms (SNPs) linked to the resistance trait (Fig. 3) but also identified flanking markers ST04_03ch05_1503657 and ST04_03ch05_6079232 that can be used to screen a larger segregating population that has been established to identify recombinant clones in the *H2* interval (Fig. 4). The relative position of the *H2* resistance genes on potato chromosome 5 is in the vicinity of the late blight resistance gene *R1* (Ballovora et al. 2002) and the Cycling Dof Factor1 (StCDF1) that controls earliness (Hannapel et al. 2017; Fig. 4).

The combination of GenSeq and RenSeq (Chen et al. 2018) targets less than 3000 genes and thereby approximately 1% of the potato genome for resequencing. The reduction in genome complexity and the resequencing of single/low-copy genes (GenSeq) or NLRs (RenSeq) can unambiguously inform the gene positions in the potato genome or represent putative candidates genes, respectively. Previous work carried out using GenSeq and RenSeq successfully mapped the late blight resistance gene *Rpi*-*ver1* from the diploid, wild, inbreeding potato species *S. verrucosum* (Chen et al. 2018) but this study confirms that this approach is also suitable for mapping in tetraploid species.

Furthermore, using the RenSeq-derived reads from the progeny parents in a diagnostic analysis (dRenSeq) rather than a mapping study confirmed that the *H2* resistance cannot be explained by previously identified genes including those associated with nematodes, viruses, or late blight resistance (Fig. 2). Whereas dRenSeq did not identify any known NLRs in P55/7, several functional NLRs, including the nematode resistance gene *Gpa2*, were identified in the susceptible variety Picasso (Armstrong et al. 2018). Although we could discount the probability that *Gpa2* significantly contributed to the resistance observed to the *G. pallida* pathotype used in this experiment, further studies may be warranted to explore whether combining the *Gpa2* and *H2* resistances has any merit. Indeed, *Gpa2* segregated in the resulting F1 progeny population and was identified, through dRenSeq, in both resistant and susceptible bulks (data not shown). Since pyramiding partially effective PCN resistance loci is an important part of resistance-breeding strategies, future studies could assess the combined effects of *Gpa2* and *H2* resistances in selected progeny clones on controlling more diverse nematode populations.

The presence of NLR *Rx* in Picasso limited the use of the PVX agro-infection assay to validate the dRenSeq data, and instead, the functionality of these genes was tested using an *Agrobacteria*-based vacuum infiltration approach. In line with dRenSeq, the transient expression of cognate pathogen effectors confirmed the functionality of the identified genes in Picasso and did not elicit a response in P55/7. This provides evidence that dRenSeq is highly robust and, as more and more genes are functionally characterised, provides a tool that can be universally applied to prioritise novel resistances against diverse pathogens including late blight and nematodes.

In this study, we have further developed the RenSeq and GenSeq analysis tools and algorithms required to map NLRs in a tetraploid configuration. Furthermore, we have shown that the RenSeq, dRenSeq and GenSeq workflows are not limited to *P. infestans* resistances and can be applied to map NLRs effective towards diverse pathogens including PCN.

## Electronic supplementary material

Below is the link to the electronic supplementary material.

Supplementary material 1 (DOCX 549 kb)

Supplementary material 2 (XLSX 11 kb)

Supplementary material 3 (XLSX 20 kb)

Supplementary material 4 (XLSX 11 kb)

Supplementary material 5 (XLSX 10 kb)

Supplementary material 6 (XLSX 10 kb)

Supplementary material 7 (XLSX 11 kb)

## References

[CR1] Andrews S (2010) FastQC: a quality control tool for high throughput sequence data. http://www.bioinformatics.babraham.ac.uk/projects/fastqc

[CR2] Armstrong MR, Vossen J, Tze-Yin Lim J, Hutten RCB, Jianfei X, Strachan SM, Harrower B, Champouret N, Gilroy EM, Hein I (2018). Tracking disease resistance deployment in potato breeding by enrichment sequencing. Plant Biotechnol J.

[CR3] Aronesty E (2011) ea-utils: command-line tools for processing biological sequencing data. https://github.com/ExpressionAnalysis/ea-utils

[CR4] Ballvora A, Ercolano MR, Weiss J, Meksem K, Bormann CA, Oberhagemann P, Salamini F, Gebhardt C (2002). The *R1* gene for potato resistance to late blight (*Phytophthora infestans*) belongs to the leucine zipper/NBS/LRR class of plant resistance genes. Plant J.

[CR5] Bendahmane A, Köhm BA, Dedi C, Baulcombe DC (1995). The coat protein of potato virus X is a strain-specific elicitor of Rx1-mediated virus resistance in potato. Plant J.

[CR6] Blok VC, Phillips MS (2012). Biological characterisation of *Globodera pallida* from Idaho. Nematology.

[CR7] Blok VC, Phillips MS, Harrower BE (1997). Comparison of British populations of potato cyst nematodes with populations from continental Europe and South America using RAPDs. Genome.

[CR8] Bos JI, Chaparro-Garcia A, Quesada-Ocampo LM, Gardener BBM, Kamoun S (2009). Distinct amino acids of the *Phytophthora infestans* effector AVR3a condition activation of R3a hypersensitivity and suppression of cell death. Mol Plant Microbe Interact.

[CR9] Bryan GJ, McLean K, Pande B, Purvis A, Hackett CA, Bradshaw JE, Waugh R (2004). Genetical dissection of H3-mediated polygenic PCN resistance in a heterozygous autotetraploid potato population. Mol Breed.

[CR10] Camacho C, Coulouris G, Avagyan V, Ma N, Papadopoulos J, Bealer K, Madden TL (2009). BLAST+: architecture and applications. BMC Bioinform.

[CR11] Caromel B, Gebhardt C (2011). Breeding for nematode resistance: use of genomic information. Genomics and molecular genetics of plant-nematode interactions.

[CR12] Chapman S, Stevens LJ, Boevink PC, Engelhardt S, Alexander CJ, Harrower B, Champouret N, McGeachy K, Van Weymers PSM, Chen X, Birch PRJ, Hein I (2014). Detection of the virulent form of AVR3a from *Phytophthora infestans* following artificial evolution of potato resistance gene *R3a*. PLoS ONE.

[CR13] Chen X, Lewandowska D, Armstrong MR, Baker K, Lim T-Y, Bayer M, Harrower B, McLean K, Jupe F, Witek K (2018). Identification and rapid mapping of a gene conferring broad-spectrum late blight resistance in the diploid potato species *Solanum verrucosum* through DNA capture technologies. Theor Appl Genet.

[CR14] Crampton BG, Hein I, Berger DK (2009). Salicylic acid confers resistance to a biotrophic rust pathogen, *Puccinia substriata*, in pearl millet (*Pennisetum glaucum*). Mol Plant Pathol.

[CR15] Davies LJ, Elling AA (2015). Resistance genes against plant-parasitic nematodes: a durable control strategy?. Nematology.

[CR16] Dunnett J (1961) Inheritance of resistance to potato root eelworm in a breeding line stemming from *Solanum multidissectum* Hawkes. In: Report of the scottish plant breeding station, pp 39–46

[CR17] Ellenby C (1954). Tuber forming species and varieties of the genus Solanum tested for resistance to the potato root eelworm *Heterodera rostochiensis* Wollenweber. Euphytica.

[CR18] Ganal M, Simon R, Brommonschenkel S, Arndt M, Phillips M, Tanksley S, Kumar A (1995). Genetic mapping of a wide spectrum nematode resistance gene (Hero) against *Globodera rostochiensis* in tomato. Mol Plant Microbe Interact MPMI.

[CR19] Gebhardt C, Mugniery D, Ritter E, Salamini F, Bonnel E (1993). Identification of RFLP markers closely linked to the H1 gene conferring resistance to *Globodera rostochiensis* in potato. Theor Appl Genet.

[CR20] Gilroy EM, Breen S, Whisson SC, Squires J, Hein I, Kaczmarek M, Turnbull D, Boevink PC, Lokossou A, Cano LM (2011). Presence/absence, differential expression and sequence polymorphisms between PiAVR2 and PiAVR2-like in *Phytophthora infestans* determine virulence on R2 plants. New Phytol.

[CR21] Haas BJ, Kamoun S, Zody MC, Jiang RHY, Handsaker RE, Cano LM, Grabherr M, Kodira CD, Raffaele S, Torto-Alalibo T, Bozkurt TO, Ah-Fong AMV, Alvarado L, Anderson VL, Armstrong MR, Avrova A, Baxter L, Beynon J, Boevink PC, Bollmann SR, Bos JIB, Bulone V, Cai G, Cakir C, Carrington JC, Chawner M, Conti L, Costanzo S, Ewan R, Fahlgren N, Fischbach MA, Fugelstad J, Gilroy EM, Gnerre S, Green PJ, Grenville-Briggs LJ, Griffith J, Grünwald NJ, Horn K, Horner NR, Hu C-H, Huitema E, Jeong D-H, Jones AME, Jones JDG, Jones RW, Karlsson EK, Kunjeti SG, Lamour K, Liu Z, Ma L, MacLean D, Chibucos MC, McDonald H, McWalters J, Meijer HJG, Morgan W, Morris PF, Munro CA, O’Neill K, Ospina-Giraldo M, Pinzón A, Pritchard L, Ramsahoye B, Ren Q, Restrepo S, Roy S, Sadanandom A, Savidor A, Schornack S, Schwartz DC, Schumann UD, Schwessinger B, Seyer L, Sharpe T, Silvar C, Song J, Studholme DJ, Sykes S, Thines M, van de Vondervoort PJI, Phuntumart V, Wawra S, Weide R, Win J, Young C, Zhou S, Fry W, Meyers BC, van West P, Ristaino J, Govers F, Birch PRJ, Whisson SC, Judelson HS, Nusbaum C (2009). Genome sequence and analysis of the Irish potato famine pathogen *Phytophthora infestans*. Nature.

[CR22] Hannapel DJ, Sharma P, Lin T, Banerjee AK (2017). The multiple signals that control tuber formation. Plant Physiol.

[CR23] Jiang R, Li J, Tian Z, Du J, Armstrong M, Baker K, Tze-Yin Lim J, Vossen JH, He H, Portal L (2018). Potato late blight field resistance from QTL dPI09c is conferred by the NB-LRR gene R8. J Exp Bot.

[CR24] Johnson DT (2001). Will human food supply meet the need in 2025? A global and pacific regional analysis. J S Pac Agric.

[CR25] Jupe F, Pritchard L, Etherington GJ, MacKenzie K, Cock PJ, Wright F, Sharma SK, Bolser D, Bryan GJ, Jones JD (2012). Identification and localisation of the NB-LRR gene family within the potato genome. BMC Genom.

[CR26] Jupe F, Witek K, Verweij W, Śliwka J, Pritchard L, Etherington GJ, Maclean D, Cock PJ, Leggett RM, Bryan GJ, Cardle L, Hein I, Jones JDG (2013). Resistance gene enrichment sequencing (RenSeq) enables reannotation of the NB-LRR gene family from sequenced plant genomes and rapid mapping of resistance loci in segregating populations. Plant J.

[CR27] Koboldt DC, Zhang Q, Larson DE, Shen D, McLellan MD, Lin L, Miller CA, Mardis ER, Ding L, Wilson RK (2012). VarScan 2: somatic mutation and copy number alteration discovery in cancer by exome sequencing. Genome Res.

[CR28] Kort J, Ross H, Rumpenhorst HJ, Stone AR (1977). International scheme for identifying and classifying pathotypes of potato cyst nematodes *Globodera rostochiensis* and *G. pallida*. Nematologica.

[CR29] Langmead B, Salzberg SL (2012). Fast gapped-read alignment with Bowtie 2. Nat Methods.

[CR30] Li H, Handsaker B, Wysoker A, Fennell T, Ruan J, Homer N, Marth G, Abecasis G, Durbin R (2009). The sequence alignment/map format and SAMtools. Bioinformatics.

[CR31] Li G, Huang S, Guo X, Li Y, Yang Y, Guo Z, Kuang H, Rietman H, Bergervoet M, Vleeshouwers VG (2011). Cloning and characterization of R3b; members of the R3 superfamily of late blight resistance genes show sequence and functional divergence. Mol Plant Microbe Interact.

[CR32] Lokossou AA, T-h P, van Arkel G, Arens M, Ruyter-Spira C, Morales J, Whisson SC, Birch PR, Visser RG, Jacobsen E (2009). Exploiting knowledge of R/Avr genes to rapidly clone a new LZ-NBS-LRR family of late blight resistance genes from potato linkage group IV. Mol Plant Microbe Interact.

[CR33] Lukasik E, Takken FL (2009). STANDing strong, resistance proteins instigators of plant defence. Curr Opin Plant Biol.

[CR34] Masler EP, Kastin AJ (2013). Free-living nematodes. Handbook of biologically active peptides.

[CR35] Milligan SB, Bodeau J, Yaghoobi J, Kaloshian I, Zabel P, Williamson VM (1998). The root knot nematode resistance gene Mi from tomato is a member of the leucine zipper, nucleotide binding, leucine-rich repeat family of plant genes. Plant Cell.

[CR36] Nicol JM, Turner SJ, Coyne DL, den Nijs L, Hockland S, Tahna Maafi Z, Jones JGGF (2010). Current nematode threats to world agriculture. Genomics and molecular genetics of plant-nematode interactions.

[CR37] Paal J, Henselewski H, Muth J, Meksem K, Menéndez CM, Salamini F, Ballvora A, Gebhardt C (2004). Molecular cloning of the potato Gro1-4 gene conferring resistance to pathotype Ro1 of the root cyst nematode *Globodera rostochiensis*, based on a candidate gene approach. Plant J.

[CR38] PGSC (2011). Genome sequence and analysis of the tuber crop potato. Nature.

[CR39] Picard D, Sempere T, Plantard O (2007). A northward colonisation of the Andes by the potato cyst nematode during geological times suggests multiple host-shifts from wild to cultivated potatoes. Mol Phylogenetics Evol.

[CR40] Quinlan AR, Hall IM (2010). BEDTools: a flexible suite of utilities for comparing genomic features. Bioinformatics.

[CR41] R Core T (2018) R: a language and environment for statistical computing, Vienna, Austria. R Foundation for Statistical Computing. http://www.R-project.org/

[CR42] RStudio (2014) RStudio: integrated development for R. RStudio, Inc., Boston, MA. http://www.rstudio.com/

[CR43] Sacco MA, Koropacka K, Grenier E, Jaubert MJ, Blanchard A, Goverse A, Smant G, Moffett P (2009). The cyst nematode SPRYSEC protein RBP-1 elicits Gpa2- and RanGAP2-dependent plant cell death. PLoS Pathog.

[CR44] Sharma SK, Bolser D, de Boer J, Sønderkær M, Amoros W, Carboni MF, D’Ambrosio JM, de la Cruz G, Di Genova A, Douches DS (2013). Construction of reference chromosome-scale pseudomolecules for potato: integrating the potato genome with genetic and physical maps. G3: Genes Genomes Genet.

[CR45] Twining S, Clarke J, Cook S, Ellis S, Gladders P, Ritchie F, Wynn S (2009). Pesticide availability for potatoes following revision of directive 91/414/EEC: impact assessments and identification of research priorities.

[CR46] Urbach JM, Ausubel FM (2017). The NBS-LRR architectures of plant R-proteins and metazoan NLRs evolved in independent events. Proc Natl Acad Sci USA.

[CR47] van der Voort JR, Kanyuka K, van der Vossen E, Bendahmane A, Mooijman P, Klein-Lankhorst R, Stiekema W, Baulcombe D, Bakker J (1999). Tight physical linkage of the nematode resistance gene Gpa2 and the virus resistance gene Rx on a single segment introgressed from the wild species *Solanum tuberosum* subsp. *andigena* CPC 1673 into cultivated potato. Mol Plant Microbe Interact.

[CR48] van der Voort JR, van der Vossen E, Bakker E, Overmars H, van Zandroort P, Hutten R, Lankhorst RK, Bakker J (2000). Two additive QTLs conferring broad-spectrum resistance in potato to *Globodera pallida* are localized on resistance gene clusters. Theor Appl Genet.

[CR49] Van Weymers PS, Baker K, Chen X, Harrower B, Cooke DE, Gilroy EM, Birch PR, Thilliez GJ, Lees AK, Lynott JS (2016). Utilizing “Omic” technologies to identify and prioritize novel sources of resistance to the oomycete pathogen *Phytophthora infestans* in potato germplasm collections. Front Plant Sci.

[CR50] Wei CF, Kvitko BH, Shimizu R, Crabill E, Alfano JR, Lin NC, Martin GB, Huang HC, Collmer A (2007). A *Pseudomonas syringae* pv. tomato DC3000 mutant lacking the type III effector HopQ1-1 is able to cause disease in the model plant *Nicotiana benthamiana*. Plant J.

[CR51] Witek K, Jupe F, Witek AI, Baker D, Clark MD, Jones JD (2016). Accelerated cloning of a potato late blight–resistance gene using RenSeq and SMRT sequencing. Nat Biotechnol.

[CR52] Zhang C, Liu L, Wang X, Vossen J, Li G, Li T, Zheng Z, Gao J, Guo Y, Visser RG (2014). The Ph-3 gene from *Solanum pimpinellifolium* encodes CC-NBS-LRR protein conferring resistance to *Phytophthora infestans*. Theor Appl Genet.

