# Supplementary material for: Mapping the H2 resistance effective against Globodera pallida pathotype Pa1 in tetraploid potato
Source: Theor Appl Genet. 2019 Jan 21;132(4):1283–94. doi: 10.1007/s00122-019-03278-4 (PMC6449323; doi:10.1007/s00122-019-03278-4)

**Fig. S1:** Vacuum infiltrated Picasso (A) and P55/7 (B) potato leaves. Cognate effectors *Avr3a^KI^, Avr3b,* PVX *coat protein (Cp),* and *RBP1* were infiltrated in order to visualise a cell death response if the corresponding *R* gene was present. *eGFP* and *Avr2* were used as negative controls. The cell death inducer CRN2 was used as a positive control. Responses were recorded 10 days after infiltration.
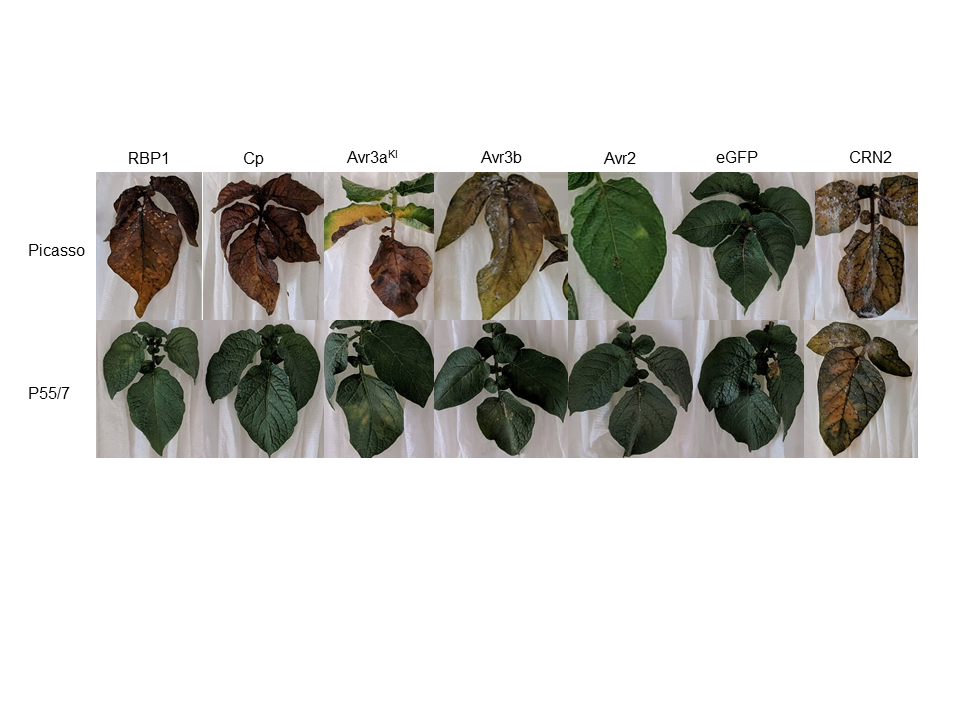

Supplement: Supplementary file 1 — Supplementary material 1 (DOCX 549 kb) [file 122_2019_3278_MOESM1_ESM.docx]
